# Preparation of Induced Pluripotent Stem Cells Using Human Peripheral Blood Monocytes

**DOI:** 10.1089/cell.2018.0024

**Published:** 2018-12-03

**Authors:** Sumito Isogai, Naoki Yamamoto, Noriko Hiramatsu, Yasuhiro Goto, Masamichi Hayashi, Masashi Kondo, Kazuyoshi Imaizumi

**Affiliations:** ^1^ Department of Respiratory Medicine, Fujita Health University School of Medicine, Toyoake, Japan.; ^2^ Regenerative Medicine Support Promotion Facility, Fujita Health University Center for Research Promotion and Support, Toyoake, Japan.

**Keywords:** human monocytes, induced pluripotent stem cells, self-renewal, three germ layers

## Abstract

Since induced pluripotent stem (iPS) cells have been established, in recent years, clinical transplantation of cells differentiated from iPS cells derived from human skin fibroblasts is been in progress. On the contrary, monocytes have complete genome information without damage and gene recombination, they are contained in the peripheral blood by ∼3%–8% and differentiate into dendritic cells that are the type of control tower for immune cells. However, generation of monocyte-derived iPS cells has only been successful when special persistent Sendai virus vectors have been used. Therefore, in this study, as a preculture method for monocytes, a culture method for maintaining activity without using any cytokine was established, and using a commercially available vector without genetic toxicity without damaging the chromosome of the cell, iPS cells derived from monocytes were successfully produced. This cell has the ability to differentiate into three germ layers, and when compared with commercially available iPS cells, there was no significant difference between self-renewal and gene expression in the three germ layers. In future, we will compare the differentiation induction of monocyte-derived iPS cells with dendritic cells and investigate the production of dendritic cells that can cope with various antigens.

## Introduction

Induced pluripotent stem (iPS) cells transfected with defined transcription factors and derived from fibroblast were first generated from mouse cells (Takahashi and Yamanaka, 2006) and human cells (Takahashi et al., 2007). The iPS cells show the same properties as embryonic stem (ES) cells, such as pluripotency, with the ability to differentiate into cells that constitute various organs. iPS cells are attracting attention in a wide range of research fields, including regeneration therapy directed at clinical application of cell transplantation, elucidation of pathological conditions, and drug discovery.

For example, in 2014, in an effort to expedite clinical application of iPS cells, Takahashi et al. induced the differentiation of iPS cells derived from skin fibroblasts from patients with age-related macular degeneration (AMD) to produce a sheet of retinal pigment epithelial cells that was surgically transplanted as an autograft into the subretinal space of the AMD patient (Mandai et al., 2017).

A premise for iPS cells is that they possess pluripotency to differentiate into ectodermal, mesodermal, and endodermal cells. Kim et al. (2010) report that the differentiation capacity of iPS cells to develop into the three germ layers depends on the type of starting cells because iPS cells are affected during the reprogramming process by the epigenetic memory of the original cells.

On the contrary, Nishizawa et al. (2016) advocated that the difference in pluripotency of iPS cells by cloning does not depend on the type of starting cells, but on the difference in DNA methylation during the reprogramming process, and thus, high-quality iPS cells with excellent pluripotency can be generated and cloned by preventing abnormal DNA methylation, regardless of the type of starting cells. However, results from Nishizawa et al. (2016) show that many clones with higher pluripotency for differentiation into blood cells are obtained from iPS cells derived from hemocytes, rather than iPS cells derived from other cell types.

Collecting ∼10 mL of peripheral blood is one useful method for collecting starting cells for iPS generation requiring minimal invasiveness. Because the monocytic genome in the nucleated cells of peripheral blood have not undergone irreversible recombination, mutation, deficiency, and so on (Nakanishi and Otsu, 2012), we focused on monocytes. However, it may be difficult to generate monocytic-derived iPS cells because maintenance of the starting cells in culture over a period of time is necessary during the reprogramming process for iPS generation, and monocytes barely proliferate *in vitro*. At present, generation of monocyte-derived iPS cells has only been successful when special persistent Sendai virus vectors (SeVdp) (Nishimura et al., 2007, 2011) developed by Nakanishi et al. have been used (Iizuka-Koga et al., 2017).

In this study, we searched for a simple monocyte culture method while maintaining cell activity, without the use of cytokines related to cell proliferation. We report the establishment of a novel, unique method for generating and culturing feeder-free human monocyte-derived iPS cells (hM-iPSCs). Commercially available vectors known to not damage the chromosomes of cultured monocytes were used and resulted in successful reprogramming of the monocytes in a timely manner.

## Materials and Methods

### Isolation and culture of human monocytes

Ten milliliters of human peripheral blood from a healthy volunteer was centrifuged at 4°C, 1400 × *g* for 10 minutes to purify the buffy coat (leukocyte layer). The buffy coat was overlaid on OptiPrep™ (Alere Technologies AS, Oslo, Norway) density-gradient media that had been adjusted to a specific gravity of 1.077 g/cm^3^. This was centrifuged at 20°C, 800 × *g* for 20 minutes to separate the mononuclear cells. After washing twice with phosphate-buffered saline (PBS), the mononuclear cells were incubated with anti-human CD14-FITC-labeled antibody and anti-human CD19-PE-labeled antibody (Thermo Fisher Scientific, Inc., Waltham, MA) at 4°C for 20 minutes.

After washing, CD14^+^/CD19^−^ cells were sorted by flow cytometry (FCM) using FACSVantage SE (BD Bioscience, San Jose, CA), and collected to recover purified monocytes (Kanai et al., 2007). Cell morphology of the sorted CD14^+^/CD19^−^ cells was confirmed using Diff-Quick stain™ (Dade Behring, Inc., Deerfield, IL). All procedures were performed in compliance with the Recombinant DNA Experiment Safety Committee, Fujita Health University (DP16051).

### Preparation of iPS cells

Purified monocytes (2.5 × 10^5^ cells/mL) were seeded into HydroCell™ 24 multiwell plates (CellSeed, Inc., Tokyo, Japan) that suppress attachment of cells, and grow as floating cultures in 1 mL of monocyte maintenance medium consisting of RPMI-1640 (Thermo Fisher Scientific, Inc.), 10% fetal bovine serum (FBS), 2 mM GlutaMax (Thermo Fisher Scientific, Inc.), and Antibiotic antimycotic solution (Sigma-Aldrich Co., LLC., St. Louis, MO). Half of the monocyte maintenance medium was replaced with fresh medium on day 1 of culture.

The monocytes were infected on day 2 with the commercially available SeVdp CytoTune^®^-iPS 2.0 Reprogramming Kit (Medical & Biological Laboratories Co., Ltd., Aichi, Japan). The SeVdp kit delivers the required genes for reprogramming somatic cells into iPS cells. For 2 days after the gene transfer, one-half volume of the monocyte maintenance medium was replaced daily with fresh medium to remove any excess vector. On day 3 posttransfer, the monocytes were collected into a 1.5-mL microtube, washed with PBS, and seeded onto mouse embryonic fibroblast (MEF; Oriental Yeast Co., Ltd., Tokyo, Japan) feeder cells in cell-adherent 24 multiwell plates (BD Bioscience). The monocytes and MEFs were cocultured in 1 mL of the monocyte maintenance medium.

The MEF feeder cells were grown in 24 multiwell plates for adherent cells that had been treated with 0.1% gelatin-coating solution (Merck KGaA, Billerica, MA). The MEF culture medium consisted of Dulbecco's modified Eagle medium (DMEM) with 4.5 g/L d-glucose, 1 mM sodium pyruvate, 2 mM GlutaMax (Thermo Fisher Scientific, Inc.), penicillin–streptomycin solution (Sigma-Aldrich Co. LLC.), and 10% FBS. One day before seeding of the vector-treated monocytes, 10 μg/mL mitomycin-C solution (Wako Pure Chemical Industries, Ltd., Osaka, Japan) was added to the MEF cultures and incubated at 37°C with 5% CO_2_ for 135 minutes to stop cell division of the MEFs and used as feeder cells.

After the transfer of the monocytes onto the feeder cultures, the monocyte maintenance medium was changed daily until day 7 after gene transfer, at which time the medium was exchanged with Primate ES Cell Medium (ReproCELL, Inc., Kanagawa, Japan) supplemented with 5 ng/mL basic fibroblast growth factor (bFGF; ReproCELL, Inc.). The Primate ES medium was changed daily. Three weeks after monocytes were initially treated with the virus vector, when four iPS-like colonies per well were observed, the cell cultures were treated with an enzymatic cell-detachment solution containing a mixture of TrypLE™ Select (Thermo Fisher Scientific, Inc.) and Accutase^®^ (Innovative Cell Technologies, Inc., San Diego, CA) at a 1:1 ratio.

The cells with the detachment solution were incubated at 37°C for 3 minutes to allow the cells to release from the plates. The detached cells were washed with PBS, and subcultured on new monolayers of freshly prepared MEFs in Primate ES medium. At 24 hours after seeding, 10 nmol/L of Y-27632 Solution (Wako) was added to the medium. On day 2 of subculture, the medium was exchanged with iPS Mix Medium, which consisted of Primate ES Cell Medium combined with an equal volume of 10 ng/mL bFGF containing Essential 8 Medium (Thermo Fisher Scientific, Inc.).

**FIG. 1.**
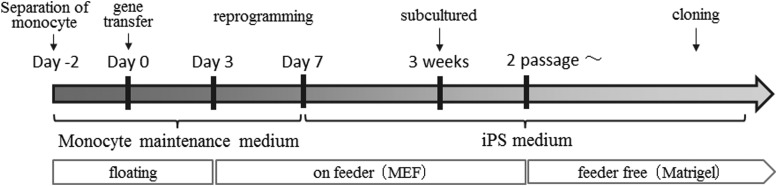
Culture flowchart from the collection and preparation of monocytes to the generation of iPS cells. iPS, induced pluripotent stem.

The iPS Mix Medium was replaced with fresh medium daily. One week after the first passage, the second passage was carried out in the same method. After a second subculture, the iPS Mix Medium was exchanged with a feeder-free culture substrate, Corning^®^ Matrigel^®^ hESC-qualified matrix (Corning Inc., Corning, NY) and only Essential 8 Medium was used for maintaining the cell cultures from 2 days after the second subculture. A schematic outline of the culture protocol is given in Fig. 1. For cloning of any iPS cells, the monocyte–MEF feeder cells just described were seeded at a density of 1 × 10^4^ cells/mL in 96-well CellBIND plates (Corning Inc.), and cultured using Essential 8 Medium.

For cloning, cells were seeded in 96 wells, cultured in the same medium for 7 days, and subcultured in the same method. This was repeated two times for cloning. The cloned transfected cells were evaluated as potential iPS cells. The culture method from monocyte to iPS cell establishment is summarized in the flowchart (Fig. 1).

### Alkaline phosphatase staining

The cloned cells were cultured in Matrigel-coated cell-adherent 12 multiwell plates (BD Bioscience) to allow formation of colonies. After fixation, the cells were subjected to alkaline phosphatase staining that is a common marker highly expressed in all pluripotent stem cells including ES cells and iPS cells, at room temperature (20°C), in the dark for 10 minutes using Red-Color™ AP Staining Kit (System Biosciences, Inc., Palo Alto, CA) following the manufacture protocol and observed.

### Quantitative reverse transcription polymerase chain reaction

The monocytes sorted from human peripheral blood before gene transfer for generation of iPS cells, and the cloned candidate iPS cells were collected separately during the appropriate phases of the studies. Total RNA was extracted from the cells using a TaqMan^®^ Gene Expression Cells-to-CT™ Kit (Thermo Fisher Scientific, Inc.). The concentrations of total RNA were measured using a NanoVue spectrophotometer (GE Health care United Kingdom Ltd, Amersham Place, United Kingdom) and the concentrations adjusted to 100 ng/μL at sterile water.

The total RNA was used as a template for reverse transcription using a GeneAmp^®^ polymerase chain reaction (PCR) System 9700 (Thermo Fisher Scientific, Inc.) thermal cycler to synthesize complementary DNA (cDNA). The cDNA was then used as template for PCR amplification. According to the protocol for TaqMan real-time PCR (quantitative PCR, qPCR) assays, qPCR was performed using an ABI PRISM 7900 HT Sequence Detection System (Thermo Fisher Scientific, Inc.) with Primer and Probe for the iPS cell-marker genes, *SOX2* (Hs00415716_m1), *OCT3/4* (Hs00742896_s1), *NANOG* (Hs04260366_g1), *KLF4* (Hs00358836_m1), and *c-MYC* (Hs00153408_m1), as well as *GAPDH* (Hs99999905_m1) as an internal positive control.

The relative expression of the monocytes before gene transfer for iPS generation and the cloned cells after gene transfer was analyzed by the delta–delta Ct method using Ct values obtained from qPCR amplification.

*SOX2*, whose expression in the monocytes before gene transfer for iPS generation was below the sensitivity of detection, was subjected to RT-qPCR and the PCR products were electrophoresed on a 2% agarose gel (Agarose 21, NIPPON GENE Co., Ltd., Tokyo, Japan) supplemented with 1 μg/mL ethidium bromide solution (Sigma-Aldrich Co. LLC.). A sample diluted six-fold with bromophenol blue loading dye (Bio Dynamics Laboratory, Inc., Tokyo, Japan) was electrophoresed at 1000 V for 40 minutes using a Mupid^®^-exU (Mupid Co., Ltd., Tokyo, Japan), and visualized using Printgraph (model AE-6911; ATTO Corporation, Tokyo, Japan).

### Immunofluorescence staining

The cloned cells were cultured in Matrigel-coated 12-well plates (BD Bioscience) and allowed to form colonies. After the cells were fixed in the multiwell plates in 10% neutral-buffered formalin at room temperature for 10 minutes, 0.5% Triton^®^ X-100 (Wako Pure Chemical Industries) was added to the wells and incubated at room temperature for 5 minutes. Subsequently, Protein Block Serum-Free Ready-To-Use (Agilent Technologies Co. Ltd., Santa Clara, CA) was added for blocking at room temperature for 5 minutes.

To this, anti-human SOX2 (an intranuclear iPS marker protein) rat monoclonal antibody (1:100; Thermo Fisher Scientific, Inc.), anti-human OCT3/4 (an intranuclear iPS marker protein), rabbit polyclonal antibody (1:500; Medical & Biological Laboratories), or anti-human NANOG (an intranuclear iPS marker protein) mouse monoclonal antibody (1:100; Thermo Fisher Scientific, Inc.) was added as primary antibody and incubated at 37°C for 1 hour. Alexa Fluor^®^ 594 labeled anti-rat immunoglobulin G (IgG) goat monoclonal antibody, Alexa Fluor 594 labeled anti-mouse IgG donkey monoclonal antibody, or Alexa Fluor 594 labeled anti-rabbit IgG goat monoclonal antibody (1:500; Thermo Fisher Scientific, Inc.) was added as secondary antibody and incubated at 37°C for 1 hour.

The immunostaining was evaluated using a fluorescence microscope (Power IX-71 and DP-71; Olympus, Tokyo, Japan).

### FCM analysis

The cloned cells (1 × 10^6^ cells/mL) were analyzed by FCM. Either anti-human SSEA-4 (an iPS cytoplasmic protein marker) mouse monoclonal antibody or anti-human TRA-1-60 (an iPS cytoplasmic protein marker) mouse monoclonal (IgM) antibody (1:100; Abcam plc., Cambridge, United Kingdom) was added to the cells as the primary antibody and incubated at 4°C for 20 minutes. As secondary antibody, Alexa Fluor 488 labeled anti-mouse IgG goat monoclonal antibody, or Alexa Fluor 488 labeled anti-mouse IgM goat monoclonal antibody (1:500; Thermo Fisher Scientific, Inc.) was added and incubated at 4°C for 20 minutes. An FACScan (Becton Dickinson) was used for cytometric analysis. To remove the dead cells from the sorting, 1 μg/mL of propidium iodide (Thermo Fisher Scientific, Inc.) was added to the samples.

### Formation of embryoid body

To confirm the pluripotency of the gene-transferred cells, we attempted to generate embryoid bodies (EBs). After cloning, 1 × 10^6^ cells were seeded into 24-well multiplates for floating cells and cultured in 1 mL of EB medium consisting of 2 mM GlutaMax, MEM nonessential amino acids solution, KnockOut™ DMEM/F12, 10% KnockOut Serum Replacement (Thermo Fisher Scientific, Inc.), StemSure^®^ 2-mercaptoethanol solution (Wako Pure Chemical Industries), and penicillin–streptomycin (Sigma-Aldrich Co. LLC.). At 24 hours after cell seeding, 5 nmol/L of Y-27632 (Wako Pure Chemical Industries) was added to the medium in each well. On the following day, 1 mL of EB medium was added. Subsequently, 1 mL of the EB medium was changed every 2 days.

### Immunostaining of EB for markers of the three germ layers

The EBs were collected from the floating cultures on day 10, placed into 2 mL microtubes, and fixed with 10% neutral-buffered formalin overnight at room temperature. The fixed EBs were dehydrated in 100% ethanol (Muto Pure Chemicals Co. Ltd., Tokyo, Japan) with the ethanol being changed every 2 hours for a total of five times. Xylene (Wako Pure Chemical Industries) was then added and changed every 2 hours for a total of three times. The final xylene was removed and the samples were placed in a heating block (Thermo block ND-M11; Nissinrika Co. Ltd., Tokyo, Japan) at 70°C, to which dissolved melting paraffin (Sigma-Aldrich Co. LLC.) was added to embed the samples. The melting paraffin was changed every 2 hours for a total of three times to produce a solid-paraffin block containing the EBs.

The paraffin-embedded EB samples were serial sectioned at a thickness of 3 μm using a microtome, processed and stained with hematoxylin/eosin (hematoxylin and eosin staining), and examined using light microscopy for morphological analysis. Next, sectioned EB specimens were subjected to immunostaining for marker proteins of the three germ layers. After deparaffinization, 0.3% hydrogen peroxide in methanol was added at room temperature for 30 minutes to inactivate endogenous peroxidase.

The specimens were immersed in citrate buffer, pH 6.0, and subjected to antigen activation at 90°C for 40 minutes using a microwave rapid immunostaining apparatus (MI-77; Azumaya Corporation, Tokyo, Japan). Protein Block Serum-Free Ready-To-Use was added at room temperature for 5 minutes for blocking. As primary antibodies, anti-human tubulin-β3 mouse monoclonal antibody (1:100; Biolegend, Inc., San Diego, CA) as an ectoderm marker protein, anti-human alpha-smooth muscle actin (α-SMA) rabbit polyclonal antibody (1:100; Abcam) as a mesoderm marker protein, and anti-human α-fetoprotein (AFP) rabbit polyclonal antibody (Proteintech Group, Inc., Rosemont, IL) as an endoderm marker protein were added and incubated at 37°C for 1 hour.

As a secondary antibody, Histofine^®^ Simple Stain™ MAX-PO MULTI (Nichirei Corporation, Tokyo, Japan) was added and incubated at 37°C for 30 minutes. Liquid 3,3′-diaminobenzidine tetrahydrochloride^+^ Substrate Chromogen System (Dako) was used as a colorimetric substrate, and the cell nuclei were stained with hematoxylin. The EB staining for the three germ layers was analyzed using a BX-51, DP-71 fluorescent upright microscope ( Olympus) equipped with a digital camera.

### Quantitative analysis of trilineage potential of the iPS cells

The self-renewal of iPS cells and gene variation related to differentiation into three germ layers were analyzed using TaqMan hPSC Scorecard™ Assay (Thermo Fisher Scientific, Inc.) (Bock et al., 2011). The iPS cells derived from the monocytes were compared with iPS cells derived from human peripheral endothelial progenitor cells (RPChiPS 771-2; ReproCELL, Inc.) and from human umbilical blood CD34^+^ progenitor cells (Human Episomal iPSC Line; Thermo Fisher Scientific, Inc.). The appearance of the three types of iPS cells and the appearance of the EBs resulting from induced differentiation for 7 days after the addition of sera were compared.

## Results

### Separation of monocytes and gene transfer

**FIG. 2.**
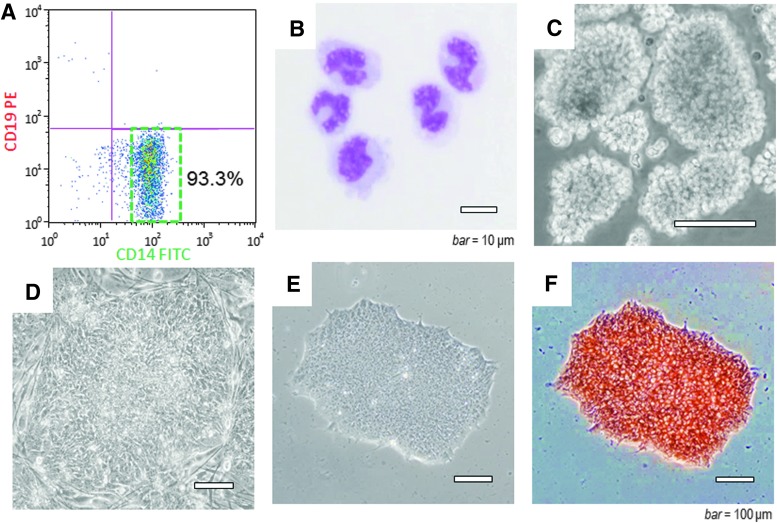
Separation of monocytes from peripheral blood and cell morphology after gene transfer. **(A)** CD14^+^/CD19^−^ cells were sorted by FCM from peripheral blood mononuclear cells to separate the monocytes. **(B)** The separated CD14^+^/CD19^−^ cells were monocytes with a size of 10 μm or larger, irregular nuclear morphology, and vacuoles (bar = 10 μm). **(C)** Floating culture for 3 days resulted in few dead cells, and most living cells forming aggregates. **(D)** Flattened iPS-cell-like colonies with clear boundaries observed on feeder cells ∼3 weeks after gene transfer. **(E)** Morphology of colonies after feeder-free culture and cloning. **(F)** All cells that formed colonies were alkaline phosphatase positive **(**scale bar in **C–F** = 100 μm**)**. FCM, flow cytometry.

Mononuclear cells were separated from human peripheral blood using a centrifugal specific gravity method, followed by cell sorting and collection of the CD14^+^/CD19^−^ (Fig. 2A) to separate pure monocytes (Fig. 2B). When the purified monocytes were subjected to floating culture, aggregates of the cells adhering to each other were formed (Fig. 2C). Cells that received the gene transfer from treatment with the SeVdp and seeded on MEF feeder cells began to form multiple colonies with the small cells aggregating within ∼2 weeks after the gene transfer.

By ∼3 weeks after transfer, flattened iPS-cell-like colonies with clear boundaries were observed (Fig. 2D). Starting from the third passage and beyond, the cultures could be maintained under feeder-free conditions, and the iPS-cell-like colonies were formed (Fig. 2E). Cloning by dilution resulted in growth rate variations among the wells. The cloned cells with the largest numbers of iPS-cell-like colonies were collected and those with the fastest growth rates after several rounds of subculture were selected. The cloned cells stained positively for alkaline phosphatase (Fig. 2F).

### Analysis of gene and protein expression

**FIG. 3.**
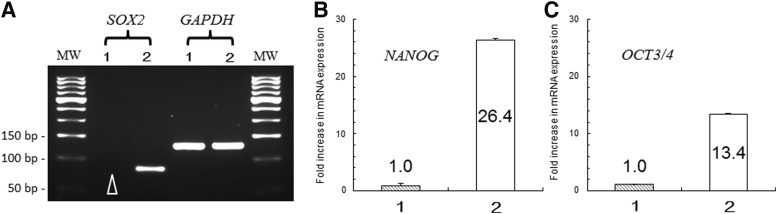
Changes in gene expression after gene transfer. **(A)** The expression of *SOX2* was not detected in the monocytes before gene transfer (*arrow head*), but was detectable in the cells after gene transfer. **(B)** When the expression level of *NANOG* in the monocytes before gene transfer was normalized to 1, the expression of *NANOG* increased 26.4-fold after the gene transfer. **(C)** Similar to the results for *NANOG*, the expression of *OCT3/4* increased 13.4-fold after the gene transfer. Sample 1 in all panels is the representative **(A)** or average **(B, C)** expression level in the monocytes before gene transfer, whereas sample 2 is the representative **(A)** or average **(B, C)** expression level in the cells after gene transfer.

Analysis by real-time PCR revealed that the expression of the SOX2 gene was below the sensitivity of detection in monocytes before the gene transfer, but *SOX2* transcripts were detected in the cells after gene transfer (Fig. 3A). The expression of *NANOG* (Fig. 3B), *OCT3/4* (Fig. 3C), *KLF4*, and *c-MYC* increased to 26.4 ± 0.3, 13.4 ± 0.2, 1.5 ± 0.2, and 6789.6 ± 0.2, respectively (increased number of expression compared with gene expression in derived cell of monocyte).

**FIG. 4.**
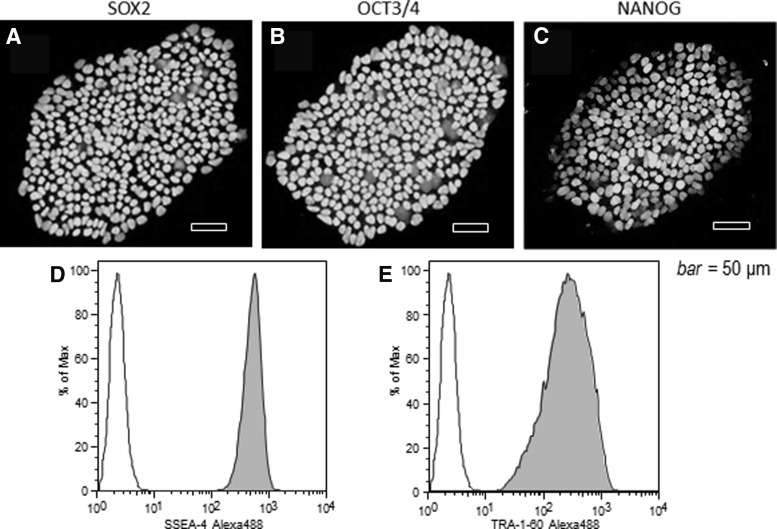
Immunostaining of cells after cloning and FCM. **(A)** Staining of the cells for the SOX2 antigen after gene transfer. **(B)** Staining for the OCT3/4 antigen. **(C)** Staining for the NANOG antigen. The cell nuclei stained positive for each antigen (scale bar = 50 μm). The expression of cell surface antigens **(D)** SSEA-4, and **(E)** TRA-1-60 was analyzed by FCM. SSEA-4 and TRA-1-60 were expressed in almost all cells.

Expression of SOX2, OCT3/4, and NANOG proteins detected by immunostaining was observed in the nucleus of the cells (Fig. 4A–C). FCM analysis indicated that SSEA-4 and TRA-1-60 were expressed in all cells (Fig. 4D, E).

### EB formation and pluripotency

**FIG. 5.**
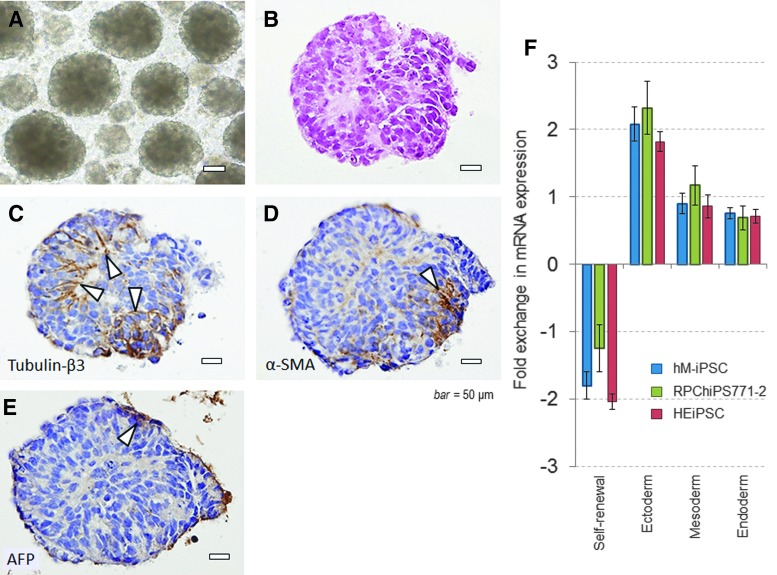
EB formation and expression of three germ layer markers. **(A)** EBs of ∼200 μm in size were formed by using a floating culture plate with EB medium. **(B)** Paraffin-embedded sections of the EBs were prepared and stained with hematoxylin and eosin and the morphology was examined using light microscopy. The cells were fully contained, even in the core of the EBs, and the cell morphology within the core of EBs was maintained. Immunostaining of EBs for **(C)** tubulin-β3, an ectoderm marker, **(D)** α-SMA, a mesoderm marker, and **(E)** anti-human α-fetoprotein, an endoderm marker, revealed positive cells at different sites (scale bar = 50 μm). **(F)** The expression level of self-renewal genes in two commercially available undifferentiated iPS cell lines, and human monocyte-derived iPS cells, and the expression level of ectodermal genes, mesodermal genes, and endodermal genes after differentiation of the cells into EBs were similar. EB, embryoid body; α-SMA, alpha-smooth muscle actin.

Multiple EBs were formed in the gene-transferred cells in floating culture for ∼10 days (Fig. 5A, B). The EBs expressed all three germ-layer protein markers, tubulin-β3, α-SMA, and AFP, with the expression of each marker being observed at a different site in the serial-sectioned paraffin-embedded EB specimens (Fig. 5C–5E).

Using a panel of genes in the Scorecard Assay, we analyzed the scores for self-renewal and those for differentiation into endoderm, mesoderm, and exoderm, respectively. It was revealed that when the iPS cells formed EBs, the expression of the genes related to self-renewal decreased, and the genes related to differentiation into the three germ layers increased for all cases (Fig. 5F). The gene expression by the hM-iPSC and two commercially available iPS cells were similar, and consistent with the results of the EB formation experiment, the ectoderm gene expression tended to be relatively high in the 7-day induced differentiation.

## Discussion

Recently, human iPS cells have been generated from several types of somatic cells including skin fibroblasts (Jaenisch and Young, 2008) and blood mononuclear cells (Chen et al., 2013; Churko et al., 2013), and have been used for research. However, in view of the clinical applications, including cell transplant therapy, it is desirable to first, avoid the risk of carcinogenesis because of residual iPS cells at the time of differentiation induction; second, to be as minimally invasive as possible to the patients during the collection of the starting cells; and third, to collect the starting cells with the least damaged genetic information and fewest mutations as a result recombination, as far as possible.

Among the peripheral blood mononuclear cells that can be collected at a relatively low level of invasiveness at usual clinical sample volumes, the number of lymphocytes is the largest. However, they are influenced by immunological reactions in our bodies, and irreversible recombination has occurred in the cell receptor genes or antibody genes. Because of this, it is reported that the iPS cells generated from lymphocytes are able to induce differentiation only to immune cells that respond to limited antigens (Loh et al., 2010; Seki et al., 2010).

Meanwhile, among the mononuclear cells, the number of stem/progenitor cells in peripheral blood is as very small, at 0.01% or less. Therefore, in the induction of iPS cells with a small volume of blood, *in vitro* proliferation culture in medium supplemented with multiple cytokines for stimulation of growth is necessary as a preliminary step. On the contrary, monocytes have complete genomic information without damage or recombination, and are contained in peripheral blood at 3%–8%. Therefore, they are more suitable as starting cells for iPS cell generation.

Monocytes are one of the immature phagocytes in peripheral blood. They exist as monocytes in the spleen and blood vessels, but in other tissues they differentiate into macrophages with potent phagocytic and antigen-presenting abilities, a type of control tower for immune cells that are involved in antigen presentation (Swirski et al., 2009). The most important point of this study was that we succeeded in keeping the survival rate of the monocytes relatively high, maintaining them without differentiation, and initiating the reprogramming at an appropriate timing after blood collection.

The cell cycle of the monocytes immediately after separation was almost in G0/G1 phase, but when they were cultured for 3 days in the monocyte maintenance medium, the monocytes viability was maintained at 90% or more, and the cell cycle began to progress slightly (data not given). In addition, because culturing monocytes in petri dishes by increasing the amount of additives leads to differentiation, the phagocytic ability increases more than that observed *in vivo* within the blood vessels. We presumed that reprogramming of monocytes was successful as the cell viability was high and maintained without differentiation in several days.

In other words, using gene transfer and reprogramming with the minimum required medium composition, and with appropriate timing, we succeeded in expressing the genes and markers detected in common iPS cells, and generated pluripotent hM-iPSCs with the ability to differentiate into all three germ layers. For reference, we used STEMCCA™ Lentivirus Reprogramming Kits (Merck KGaA), Epi5™ Episomal iPSC Reprogramming Kit (Thermo Fisher Scientific, Inc.) as other tools to reprogram, but iPS cells from human monocytes could not be produced.

Since the first paper on iPS cells (Takahashi and Yamanaka, 2006) was published in 2006, the research has been diverse, including development of iPS cell generation methods, identification of the transcription factors required for reprogramming, and efforts toward clinical application of iPS cells. While conducting research on issues such as generation efficiency of iPS cells and time required for generation, it is reported that the experimental success rate of iPS cell generation is 50% or more, and initialization is further prompted by combining *Zic3* and *Esrrb* genes for efficient generation of mouse iPS cells (Sone et al., 2017).

In addition, an experimental method for the direct generation of the cells with the desired function of the target cells was developed by introducing specific transcription factors (the four most important transcription factors: SPI1, CEBPA, MNDA, and IRF8) without the need of generating iPS cells. In fact, generation of the cells possessing some functions similar to human monocytes from skin-derived human fibroblasts was successful (Suzuki et al., 2012).

The ultimate goal of our project was to develop a novel immune cell therapy using immune cells derived from iPS cells through differentiation induction. This new type of immune cell therapy would allow for clinical conditions of refractory allergic disease such as asthma to be regulated in an antigen-specific manner. Specifically, we plan to induce differentiation of the iPS cells derived from patients into naive lymphocytes (naive T cell) and dendritic cells as immune cells (Senju et al., 2009; Yanagimachi et al., 2013), and to prepare antigen-specific regulatory T cells (Suzuki et al., 2017) by immunization with antigens according to the pathological conditions of the patients, and coculturing with IL-35, cytokine (Collison et al., 2010) that are added.

In this study, we succeeded for the first time in culturing monocytes in a relatively simple manner, and reprogramming them with commercially available vectors at the appropriate time. We have already begun experiments on the differentiation induction into monocytes (data not shown). In future studies, we will further induce differentiation of the iPS cells into macrophages and dendritic cells, and continue to analyze cell functions such as the differentiation efficiency of iPS cells derived from different cells, their phagocytic ability, and ability of antigen presentation.
